# Advancements in Wearable Sensor Technologies for Health Monitoring in Terms of Clinical Applications, Rehabilitation, and Disease Risk Assessment: Systematic Review

**DOI:** 10.2196/76084

**Published:** 2026-01-09

**Authors:** Bonsang Gu, Hyeon Su Kim, HyunBin Kim, Jun-Il Yoo

**Affiliations:** 1 Department of Biomedical Research Institute Inha University Hospital Incheon Republic of Korea; 2 Program in Biomedical Science and Engineering Inha University Incheon Republic of Korea; 3 Department of Orthopedic Surgery Inha University Hospital, Inha University College of Medicine Incheon, Incheon Republic of Korea

**Keywords:** wearable sensors, health monitoring, systematic review, gait analysis, rehabilitation, mobile health, mHealth

## Abstract

**Background:**

Wearable sensor technologies such as inertial measurement units, smartwatches, and multisensor systems have emerged as valuable tools in clinical and real-world health monitoring. These devices enable continuous, noninvasive tracking of gait, mobility, and functional health across diverse populations. However, challenges remain in sensor placement standardization, data processing consistency, and real-world validation.

**Objective:**

This systematic review aimed to evaluate recent literature on the clinical and research applications of wearable sensors. Specifically, it investigated how these technologies are used to assess mobility, predict disease risk, and support rehabilitation. It also identified limitations and proposed future research directions.

**Methods:**

This review was conducted according to the PRISMA (Preferred Reporting Items for Systematic Reviews and Meta-Analyses) guidelines. We searched the PubMed, Scopus, and Web of Science databases up to March 9, 2025. Inclusion criteria focused on studies using wearable sensors in clinical or real-world environments. A total of 30 eligible studies were identified for qualitative synthesis. Data extracted included study design, population characteristics, sensor type and placement, machine learning algorithms, and clinical outcomes.

**Results:**

Of the included studies, 43% (13/30) were observational, 27% (8/30) were experimental, and 10% (3/30) were randomized controlled trials. Inertial measurement unit–based sensors were used in 67% (20/30) of the studies, with wrist-worn devices being the most common (13/20, 65%). Machine learning techniques were frequently applied, with random forest (6/30, 20%) and deep learning (5/30, 17%) models predominating. Clinical applications spanned Parkinson disease, stroke, multiple sclerosis, and frailty, with several studies (4/30, 13%) reporting high predictive accuracy for fall risk and mobility decline (area under the receiver operating characteristic curve up to 0.97).

**Conclusions:**

Wearable sensors show strong potential for mobility monitoring, disease risk assessment, and rehabilitation tracking in clinical and real-world settings. However, challenges remain in standardizing sensor protocols and data analysis. Future research should focus on large-scale, longitudinal studies; harmonized machine learning pipelines; and integration with cloud-based health systems to improve scalability and clinical translation.

## Introduction

Throughout this paper, we use standardized terminology to ensure clarity and consistency. The term “wearable sensor” refers to any body-worn device capable of measuring physiological or biomechanical parameters, including inertial measurement unit (IMU)–based sensors, smartwatches, smart insoles, and multisensor systems. “IMU” refers specifically to devices incorporating accelerometers, gyroscopes, and magnetometers for motion tracking. When referring to specific device types (eg, smartwatches and smart insoles), we use the precise terminology to distinguish their unique features and applications.

Wearable sensors have gained significant attention in clinical research and health care for their ability to provide continuous, real-world assessments of mobility and physiological health. These devices, including IMU-based sensors, smartwatches, and multisensor systems, have transformed traditional gait and activity monitoring by enabling remote, noninvasive tracking of movement patterns and health status [1]. The integration of advanced analytics, particularly machine learning (ML), has further enhanced their diagnostic and predictive capabilities, positioning wearable sensors as key tools in digital health and precision medicine [2].

Gait analysis and mobility tracking have been central to wearable sensor applications, particularly in neurological, musculoskeletal, and age-related conditions. In Parkinson disease (PD), wearable sensors have been used to detect subtle changes in gait speed, stance and swing phase durations, and postural instability, aiding in early disease detection and progression monitoring. In stroke rehabilitation, these sensors enable remote motor recovery assessment and provide continuous mobility data outside traditional clinical settings [3]. Wearable sensors also demonstrate high efficacy in frailty assessment and fall risk prediction, offering objective, real-time alternatives to conventional tools such as the Performance-Oriented Mobility Assessment (POMA) and Timed Up and Go (TUG) tests.

Despite their growing clinical adoption, several challenges hinder the widespread implementation of wearable sensor technology. Variability in sensor placement, study methodologies, and data processing techniques limits cross-study comparability and reproducibility [3]. Additionally, while controlled laboratory studies have validated their accuracy, real-world validation remains insufficient, necessitating further large-scale, longitudinal studies to assess their usability and reliability across diverse populations [4]. Furthermore, standardization of ML frameworks and data interpretation methodologies is essential to ensure consistent clinical application [5].

This systematic review aimed to provide a comprehensive evaluation of wearable sensor research, analyzing their clinical applications, technological advancements, and methodological challenges. By synthesizing evidence from recent studies, we highlight key trends in wearable sensor use, discuss their implications for health care, and propose future directions to enhance their impact in mobility monitoring and rehabilitation.

## Methods

### Study Design

The protocol of this review was not registered in PROSPERO due to its exploratory nature and inclusion of emerging sensor studies. However, the review process followed the PRISMA (Preferred Reporting Items for Systematic Reviews and Meta-Analyses) 2020 guidance to ensure methodological transparency. The study selection process followed PRISMA 2020 guidelines, including identification, screening, eligibility assessment, and final inclusion. This review focused on studies published in the last 10 years that investigated the applications and effectiveness of wearable sensors, including smartwatches, in remote health monitoring, rehabilitation, and disease assessment. Full-text articles were included to ensure a comprehensive analysis. We aimed to synthesize evidence on clinical and research applications of wearable sensors, particularly for gait analysis, fall risk assessment, and disease monitoring. Given study heterogeneity, we categorized and synthesized the findings narratively, emphasizing disease-specific insights and sensor use trends.

### Search Strategy

A comprehensive database search was conducted across PubMed, Scopus, and Web of Science.

The search strategy combined terms related to wearable technologies, inertial sensors, digital biomarkers, and rehabilitation. Representative Boolean operators were used as follows:

(“smartwatch” OR “smart watch” OR “wearable sensor” OR “wearable sensor”) AND (“accelerometer” OR “acceleration sensor” OR “inertial sensor” OR “IMU”) AND (“remote monitoring” OR “digital biomarkers” OR “telemedicine” OR “wearable health tracking”) AND (aging OR older adults OR elderly OR Parkinson OR stroke OR “gait disorders” OR “neurological disorders” OR “movement disorders” OR “fall risk” OR rehabilitation OR “functional mobility” OR sarcopenia OR osteoarthritis OR dementia) NOT review.

The initial search yielded 4226 records. Of these 4226 records, after removing duplicates and studies unrelated to clinical applications (n=3664, 86.7%), 562 (13.3%) remained for screening. Of these 562 studies, those focusing solely on technical performance comparisons (n=501, 89.1%) were excluded, leaving 61 (10.9%) for eligibility assessment. Of these 61 studies, an additional 31 (51%) were excluded due to limited relevance to disease-related applications, resulting in 30 (49%) studies included in the final review. The search was finalized on March 9, 2025. In total, 30 studies met the inclusion criteria and were included in the final synthesis. Additional references cited throughout the manuscript (n=43) were used for background, context, and methodological justification.

### Study Selection Process

A single researcher conducted study selection and data extraction following a predefined protocol to minimize bias. The eligibility criteria were clearly defined and consistently applied. Studies published from March 9, 2015, to March 9, 2025, were considered, reflecting a 10-year search window. Eligible study designs included randomized controlled trials (RCTs), observational studies, and experimental validation studies conducted in either clinical or real-world settings. Both research-grade and commercial wearable sensors were included provided that they reported measurable health or functional outcomes. Only English-language, peer-reviewed articles were included, and conference abstracts, reviews, and purely technical feasibility reports without human participants were excluded. Quality appraisal using the Newcastle-Ottawa Scale (NOS), Joanna Briggs Institute (JBI) appraisal tools, or version 2 of the Cochrane risk-of-bias tool for randomized trials (RoB 2) was conducted to describe study rigor but did not influence inclusion decisions.

Any uncertainties during the selection process were resolved by re-evaluating studies against the predefined inclusion criteria. Data extraction was conducted manually using a standardized form. No independent reviewers cross-checked the extracted data, which is acknowledged as a limitation. Missing or unclear data were clarified when possible, by contacting the corresponding authors. No automation tools were used for data collection.

### Participant Selection in the Included Studies

The included studies targeted diverse populations, including healthy adults; older individuals; and patients diagnosed with neurological disorders (eg, stroke and PD), musculoskeletal disorders (eg, sarcopenia and osteoarthritis), or metabolic conditions (eg, diabetes). Participants could walk independently and provided informed consent. We excluded studies lacking clear participant definitions or standard gait analysis metrics to maintain consistency. Pediatric studies were excluded except for those including toddler cohorts (aged <3 years) and specifically designed for developmental gait analysis. To ensure data quality, studies were required to report a minimum wear time of 30 minutes of valid sensor data per session.

### Wearable Sensor Technology

The wearable sensors reviewed featured advanced components such as high-precision accelerometers, gyroscopes, and pressure sensors. These sensors accurately captured key gait and mobility parameters: step length, stance and swing phase durations, plantar pressure distribution, and center of pressure. Smartwatches were primarily used for activity tracking and remote monitoring, whereas wearable sensors and foot-mounted sensors specialized in gait and postural assessments. The wearable sensors integrated seamlessly into daily life, ensuring high usability and real-world applicability.

### Data Acquisition and Analysis

Data were collected using a variety of wearable sensor systems, including IMUs, smart insoles, smartwatches, and pressure-sensing devices. Sensor placement varied by study objective and included the wrist, waist, ankle, thigh, lumbar spine, and foot. The IMU sensors incorporated accelerometers, gyroscopes, and magnetometers with sampling rates ranging from 10 to 1149 Hz depending on the device and measurement context. Pressure-sensitive insoles provided additional biomechanical insights through plantar pressure distribution and force-time characteristics.

Wireless data transmission via Bluetooth or cloud platforms enabled real-time monitoring and digital biomarker extraction. Embedded preprocessing algorithms were applied to reduce noise, improve signal quality, and enhance feature extraction accuracy. Studies used various ML techniques, including random forest (6/30, 20%), deep learning (5/30, 17%), elastic net regression (4/30, 13%), and principal component analysis (PCA; 2/30, 7%), for pattern recognition, mobility classification, and disease risk prediction. All reported quantitative values (eg, area under the receiver operating characteristic curve [AUROC], accuracy, and improvement rate) were extracted from individual studies and are presented descriptively, not as pooled estimates.

The methodological quality of the included studies was systematically evaluated using appropriate assessment tools based on the study design. The NOS was applied to prospective cohort studies, whereas the JBI critical appraisal checklist was used for observational, cross-sectional, and experimental studies. For RCTs, the RoB 2 tool was used to ensure a robust evaluation of study quality. The results of the quality assessment guided the interpretation of the reliability and clinical applicability of the findings. Studies were categorized as low (JBI or NOS score of ≥8), moderate (JBI or NOS score of 6-7), or high (JBI or NOS score of ≤5) risk of bias according to established thresholds.

### Ethical Considerations

All studies included adhered to ethical standards for human research, following the Declaration of Helsinki. As this study is a systematic review of published literature and did not involve human participants, interventions, or identifiable private data, ethics approval was not required. Data privacy and participant well-being were prioritized across the studies.

## Results

### Study Design and Population Characteristics

**Figure 1:**
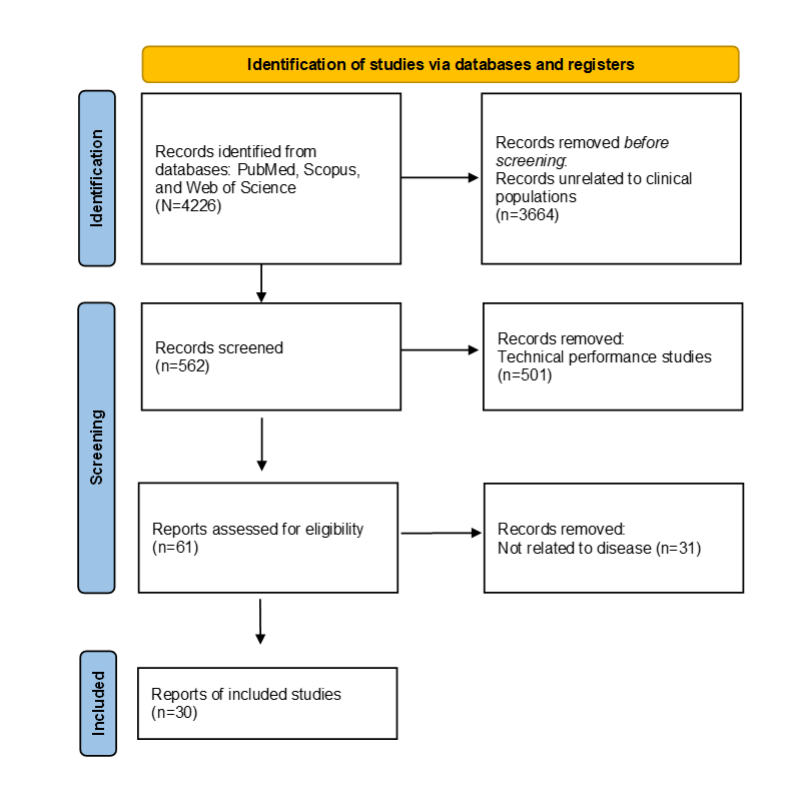
PRISMA flow diagram illustrating the screening process of papers for study selection.

A total of 4226 records were identified through database searching. After removing duplicates and screening titles and abstracts, 562 articles remained for full-text assessment. Of these, 30 studies met the inclusion criteria and were included in the final review. The full screening process is summarized in Figure 1 (PRISMA 2020 flow diagram). Among the analyzed studies, the most frequently used research design was prospective observational studies, accounting for 43% (13/30) of the total. Experimental studies comprised 27% (8/30), whereas RCTs were limited to 10% (3/30). Cross-sectional studies and cohort studies accounted for 13% (4/30) and 7% (2/30), respectively. Observational studies were most common due to their feasibility, whereas experimental studies validated sensor-based assessments. RCTs were scarce, indicating limited rigorous intervention-based evaluations. Cohort studies were included in long-term monitoring applications.

The studies focused on various populations, with healthy adults being the most common participant group, as summarized in Table 1. Studies involving neurodegenerative diseases such as PD, stroke, Huntington disease, and multiple sclerosis (MS) were also prevalent. Healthy adults were often included for sensor validation and reliability assessment, whereas studies on neurodegenerative diseases primarily aimed at mobility and functional monitoring. Research on frailty in older adults (4/30, 13%) focused on mobility assessment, balance, and fall risk analysis. The reviewed studies covered a wide range of age groups and health conditions. Studies involving healthy adults typically included participants from early adulthood to middle age, with some extending to adolescent and pediatric populations. Research on neurodegenerative diseases such as PD and Huntington disease focused primarily on older adults, whereas cardiovascular disease– and frailty-related studies involved older participants as well. Certain studies (12/30, 40%) targeted specific conditions such as osteoarthritis, rheumatoid arthritis, MS, cystic fibrosis, stroke, and spinal cord injury, highlighting the diverse application of wearable sensors, particularly smartwatches, in different clinical populations. The distribution of male and female participants varied across studies, with some (1/30, 3%) focusing specifically on age-based differences in sensor performance and health monitoring.

**Table 1:** Summary of study populations in the reviewed studies.

Study	Participant age (y)	Number of male/female participants	Health condition
Bolam et al [6], 2021	Mean 66.8 (SD 7.0)	6/8	Knee osteoarthritis
Angelucci and Aliverti [7], 2023	Mean 26.8 (SD NR^a^; range 23-54)	9/11	Healthy adults
Greene et al [8], 2021	PD^b^ 1 (clinical cohort): mean 67.3 (SD 7.1); PD 2 (exercise cohort): mean 64.9 (SD 7.3)	27/17	PD
Gordon et al [9], 2019	Mean 51 (SD 12)	9/8	HD^c^
Presley et al [10], 2023	Range 13-24	6/12	Healthy adults and adolescents
De Cannière et al [11], 2020	Mean 63 (SD 1)	65/24	CVD^d^ and heart failure
Nunes et al [12], 2024	HD: mean 51.9 (SD 11); pre-HD stage: mean 36.5 (SD 13.1); control: mean 58.9 (SD 12.2)	8/8 (HD group)	HD, pre-HD stage, and controls
Mahadevan et al [13], 2020	Control: mean 43.9 (SD 10); PD: mean 68.1 (SD 8.1)	Control: 27/33; PD: 23/12	PD
Seo et al [14], 2024	Mean 61 (SD 12)	12/7	Stroke (upper-limb hemiparesis)
Odhiambo et al [15], 2023	Mean 27.25 (SD NR; range 20-56)	16/12	Healthy adults
Hwang and Effenberg [16], 2021	Mean 29.8 (SD 6.8)	6/6	Healthy adults
Wu et al [17], 2021	Mean 48 (range 26-66)	2/2	Healthy adults
John and Soangra [18], 2022	Control: mean 74 (SD 8.7); Stroke: mean 69 (SD 8.4)	Control: 6/8; Stroke: 8/6	Stroke vs healthy older adults
Meyer et al [19], 2022	Mean 51 (SD 7)	5/16	Multiple sclerosis
Toumieux et al [20], 2015	Not specified	Not specified	Stroke
Elstub et al [21], 2022	Not specified	6/3	Healthy runners
Perraudin et al [22], 2018	RA^e^: mean 50.7 (SD 11.4); PA^f^: mean 47.5 (SD 15.5); OA^g^: mean 60.7 (SD 4.5); healthy: mean 48 (SD 13.6)	RA: 18 female; PA: 2 female; OA: 10 female; healthy: 15 female	RA, PA, and OA
Giggins et al [23], 2025	Mean 77.5 (SD 8.4)	12/39	Frailty levels
Savi et al [24], 2020	Mean 37.5 (SD 11.5)	6/18	Cystic fibrosis
Haghi et al [25], 2023	Mean 26.7 (SD NR)	22/8	Healthy adults
DasMahapatra et al [26], 2018	Mean 52 (SD 10.6)	28/86	Multiple sclerosis
Sun et al [27], 2019	OA: mean 78.2 (SD 6.1); YA^h^: mean 24.4 (SD 3.9)	OA: 4/6; YA: 6/6	Healthy adults
Ramezani et al [28], 2019	Community: mean 82.16 (SD 9.55); hospital: mean 84.22 (SD 13.87)	Community: 41/104; hospital: 5/4	Older adults with multiple chronic conditions
Liew et al [29], 2024	Mean 20.8 (SD 1.6)	9/9	Healthy adults
Kwon et al [30], 2019	13-35 months (1-year-olds: 11; 2-year-olds: 11)	10/12	Healthy toddlers
Martin et al [31], 2015	Mean 58 (SD 8; range 18-69)	26/22	CVD prevention and obesity
Hup et al [32], 2024	Various	Not specified	OHCA^i^ and SCA^j^
Browne et al [33], 2020	Mean 13.3 (SD 2.3)	9/11	Pediatric obesity
Burns et al [34], 2020	≥18	Not specified	Rotator cuff and shoulder pain
Bailey et al [35], 2024	Mean 51 (SD 9; range 28-63)	11/4	Spinal cord injury and wheelchair users

^a^NR: not reported.

^b^PD: Parkinson disease.

^c^HD: Huntington disease.

^d^CVD: cardiovascular disease.

^e^RA: rheumatoid arthritis.

^f^PA: psoriatic arthritis.

^g^OA: old adult.

^h^YA: young adult.

^i^OHCA: out-of-hospital cardiac arrest.

^j^SCA: sudden cardiac arrest.

### Sensor Use and Data Analysis in Wearable Research

Studies relied heavily on IMU sensors (20/30, 67%) and smartwatches (8/30, 27%). Shoe-mounted sensors and multisensor systems incorporating electrocardiograms were less frequently used. Wrist-worn sensors (13/30, 43%) were the most common due to ease of wear and practical data collection. Smartwatches, as a subset of wrist-worn devices, were frequently used for continuous activity tracking and health monitoring. Additionally, ankle and thigh placements (7/30, 23%) were primarily used for gait analysis, whereas foot and insole sensors (2/30, 7%) were implemented for more specialized balance and gait assessments.

The predominant activity type studied was gait analysis, which appeared in 60% (18/30) of the studies, followed by activities of daily living (12/30, 40%), balance assessments (8/30, 27%), energy expenditure evaluations (6/30, 20%), and rehabilitation exercises (6/30, 20%). Gait analysis was especially relevant in research focused on neurodegenerative diseases and mobility impairments, where smartwatches were often used for free-living gait monitoring. Studies on activities of daily living leveraged smartwatches for continuous data collection in real-world settings. Balance assessments were primarily conducted for frailty and fall risk evaluations, with some smartwatch-based apps integrating accelerometry for postural control analysis.

Data processing in wearable sensor research used a range of ML techniques. Random forest was the most commonly applied method (6/30, 20%), followed by deep learning models (5/30, 17%), elastic net regression and support vector machines (SVMs; 4/30, 13%), and PCA (2/30, 7%). Random forest was frequently used in gait analysis and activity recognition, whereas deep learning models were applied for long-term movement pattern analysis, particularly in smartwatch-based apps. Elastic net and SVM were commonly used for classification tasks, and PCA was used for dimensionality reduction, optimizing the performance of wearable sensor data processing.

### Clinical Applications of Wearable Sensors

#### Overview

The diverse clinical applications of wearable sensors, categorized into 5 main areas—healthy individuals, age-related conditions, neurological conditions, musculoskeletal disorders, and metabolic conditions—are summarized in Table 2.

**Table 2:** Summary of clinical results for wearable sensors across populations.

Category and condition		Key findings	Clinical significance and utility
**Rehabilitation assessment and functional recovery**			
	Knee arthroplasty (TKA^a^ and UKA^b^)	Bone stimulus: +52%; impact load: +371%; OKS^c^: +52%; EQ-5D: +32%	IMU^d^-based wearables support accurate postoperative monitoring and personalized recovery for knee arthroplasty.
	CR^e^	6MWD^f^ prediction error: 42.8 m; *R*^2^=0.661	CR progress can be reliably tracked remotely, improving long-term care.
	Stroke—upper-limb rehabilitation	Movement quality classification accuracy: 92%; *F*_1_-score: 0.95	IMUs enable remote monitoring of home-based rehabilitation, enhancing adherence and personalization.
	Rotator cuff injury rehabilitation	Exercise classification accuracy: 99.99%	Smartwatches improve rehabilitation compliance and exercise tracking at home.
	Lumbar mobility assessment	Significant ROM^g^ differences between wrist and lumbar sensors (up to 8, *P*=.003)	Wrist-worn sensors allow for remote lumbar mobility assessment for rehabilitation use.
**Disease state prediction and risk assessment**			
	PD^h^—tremor and fall risk	Tremor detection accuracy: 83%; sensitivity: 86%; specificity: 86%; fall risk prediction RMSE^i^: 0.42	Wearables objectively monitor PD symptoms and fall risk for early intervention.
	HD^j^	Sensitivity: 85%; specificity: 72%; accuracy: 81%; AUROC^k^: 0.82	HD motor decline can be tracked remotely for personalized care.
	Frailty assessment in older adults	QTUG^l^ accuracy: 75.8%; ScanWatch-enhanced model: 79.3%	Wearables detect frailty early, enabling preventive intervention in older adults.
	OHCA^m^ detection	Optimized balance between sensitivity and specificity	OHCA can be detected in real time via wearables for rapid emergency response.
**Activity and behavior tracking**			
	Arthritis—pain and function monitoring	5-STS^n^ performance significantly correlated with morning pain scores (*P*<.05)	IMUs enable noninvasive, remote tracking of arthritis pain and function.
	CF^o^—activity monitoring	Fitbit and iOS smartphone showed strong agreement with SWA^p^	Consumer wearables offer scalable, affordable physical activity monitoring.
	Consumer vs research-grade wearables (activity monitoring)	Z-Track sedentary behavior detection: AUC^q^=0.95; MVPA^r^ detection: AUC=0.93	Fitbit-level devices reliably track sedentary behavior and activity levels.
	Medication adherence monitoring	Medication intake detection accuracy: 93.6%; sensitivity: 92%	Wearables automate medication tracking, improving adherence in chronic care.
**Gait analysis and balance assessment**			
	Gait symmetry analysis (head-worn sensor)	Gait event detection accuracy: 99.35%	Head-worn sensors support gait symmetry analysis for neurological rehabilitation.
	Balance assessment (smartwatch based)	Strong correlation between smartwatch and research-grade sensors (*r*=0.861-0.970)	Smartwatches enable balance monitoring at home to prevent falls.
	MS^s^—balance and mobility	AUROC=0.97	Wearables track long-term mobility and balance in MS, supporting personalized rehabilitation.

^a^TKA: total knee arthroplasty.

^b^UKA: unicompartmental knee arthroplasty.

^c^OKS: Oxford knee score.

^d^IMU: inertial measurement unit.

^e^CR: cardiac rehabilitation.

^f^6MWD: 6-minute walk distance.

^g^ROM: range of motion.

^h^PD: Parkinson disease.

^i^RMSE: root mean square error.

^j^HD: Huntington disease.

^k^AUROC: area under the receiver operating characteristic curve.

^l^QTUG: quantitative timed up and go.

^m^OHCA: out-of-hospital cardiac arrest.

^n^5-STS: 5-time sit-to-stand assessment.

^o^CF: cystic fibrosis.

^p^SWA: sensewear armband.

^q^AUC: area under the curve.

^r^MVPA: moderate to vigorous physical activity.

^s^MS: multiple sclerosis.

#### Rehabilitation Assessment and Functional Recovery

Wearable sensors were used to analyze gait metrics in both young and older adults. Studies on young adults focused on plantar pressure distribution, step length, swing time, and ground reaction force, achieving high accuracy in real-time gait analysis, such as 95% using the FreeWalker system with a 1000-Hz sampling frequency. Advanced ML techniques further enhanced center of pressure prediction accuracy by over 30%. Among older adults, wearable sensors were effective in assessing fall risk and mobility. Improvements were observed in swing time (+6.45%) and slip and trip classification accuracy, which exceeded 98% (*P*<.05).

#### Disease State Prediction and Risk Assessment

Studies addressed frailty and fall history using wearable sensors to measure load distribution, gait phases, and stance and swing time. Load distribution assessments demonstrated high reliability, with intraclass correlation coefficient values reaching 0.91 and strong correlations for the left (*r*=0.7171) and right (*r*=0.7502) foot. Fall risk indexes provided significant predictive accuracy, with AUROC values of 0.919 (*P*<.05), making them comparable to traditional tools such as the POMA and TUG tests. These findings emphasize the potential of wearable sensors for early identification of frailty and fall risk in older adults.

#### Activity and Behavior Tracking

Wearable sensors were used to evaluate gait characteristics in individuals with stroke, MS, and PD. Among stroke survivors, significant reductions in gait speed and step length were observed compared to controls, with strong correlations between Fugl-Meyer Assessment lower-limb scores and stance time differences (*R*^2^=0.71). In MS, high agreement was reported between the FeetMe and GAITRite systems (intraclass correlation coefficient>0.8), validating the utility of wearable sensors for mobility monitoring. For individuals with PD, significant differences were detected in gait speed, stride length, and swing and stance time compared to healthy controls (*P*<.05), demonstrating the role of wearable sensors in tracking disease progression.

#### Gait Analysis and Balance Assessment

Wearable sensors were effective in managing diabetes and other metabolic disorders. For diabetes, total contact casts reduced forefoot contact area by 5% and peak pressure by 8% (*P*<.05), effectively offloading pressure and reducing the risk of complications. These devices provide actionable data that support better management of metabolic health and reduce disease-related complications.

### Quality Assessment Results

The quality assessment results are summarized in Table 3, with the studies rated using the JBI critical appraisal tools scoring between 5 and 8 out of 10, NOS-rated cohort studies scoring between 6 and 7 out of 9, and RoB 2–rated RCTs scoring 8 out of 10. Of the 30 included studies, 6 (20%) were rated as low risk, and 24 (80%) were rated as having a moderate risk of bias. Studies investigating wearable sensor–based mobility assessments, gait analysis, and rehabilitation applications showed high feasibility and reliability, particularly those incorporating real-time monitoring and signal processing techniques. However, several studies (26/30, 87%) exhibited limitations such as small sample sizes, lack of validation in real-world settings, and limited applicability to diverse patient populations. Additionally, some studies (12/30, 40%) faced technical challenges, including sensor displacement errors, signal-to-noise ratio issues, and data synchronization difficulties.

**Table 3:** Quality assessment summary of the reviewed studies.

Study	Study design	Quality score	Risk-of-bias category
Bolam et al [6], 2021	Prospective cohort study	7/9 (NOS^a^)	Moderate
Angelucci and Aliverti [7], 2023	Experimental study	6/10 (JBI^b^ tool)	Moderate
Greene et al [8], 2021	Experimental study	7/10 (JBI tool)	Moderate
Gordon et al [9], 2019	Observational study	6/10 (JBI tool)	Moderate
Presley et al [10], 2023	Experimental study	8/10 (JBI tool)	Low
De Cannière et al [11], 2020	Prospective cohort study	7/9 (NOS)	Moderate
Nunes et al [12], 2024	Observational study	6/10 (JBI tool)	Moderate
Mahadevan et al [13], 2020	Observational study	8/10 (JBI tool)	Low
Seo et al [14], 2024	Observational study	7/10 (JBI tool)	Moderate
Odhiambo et al [15], 2023	Experimental study	8/10 (JBI tool)	Low
Hwang and Effenberg [16], 2021	Observational study	7/10 (JBI tool)	Moderate
Wu et al [17], 2021	Observational study	7/10 (JBI tool)	Moderate
John and Soangra [18], 2022	Observational study	6/10 (JBI tool)	Moderate
Meyer et al [19], 2022	Observational study	7/10 (JBI tool)	Moderate
Toumieux et al [20], 2015	Experimental study	5/10 (JBI tool)	Moderate
Elstub et al [21], 2022	Experimental study	7/10 (JBI tool)	Moderate
Perraudin et al [22], 2018	Observational study	7/10 (JBI tool)	Low
Giggins et al [23], 2025	Cross-sectional study	6/10 (JBI tool)	Moderate
Savi et al [24], 2020	Cross-sectional study	7/10 (JBI tool)	Moderate
Haghi et al [25], 2023	Experimental study	8/10 (JBI tool)	Moderate
DasMahapatra et al [26], 2018	Observational study	6/10 (JBI tool)	Low
Sun et al [27], 2019	Experimental study	7/10 (JBI tool)	Moderate
Ramezani et al [28], 2019	Pilot study	6/10 (JBI tool)	Moderate
Liew et al [29], 2024	Cross-sectional study	6/10 (JBI tool)	Moderate
Kwon et al [30], 2019	Observational study	7/10 (JBI tool)	Moderate
Martin et al [31], 2015	RCT^c^	8/10 (RoB 2^d^)	Low
Hup et al [32], 2024	Clinical study	7/10 (JBI tool)	Moderate
Browne et al [33], 2020	RCT	8/10 (RoB 2)	Moderate
Burns et al [34], 2020	Prospective cohort study	7/9 (NOS)	Moderate
Bailey et al [35], 2024	Cross-sectional study	7/10 (JBI tool)	Moderate

^a^NOS: Newcastle-Ottawa Scale.

^b^JBI: Joanna Briggs Institute.

^c^RCT: randomized controlled trial.

^d^RoB 2: version 2 of the Cochrane risk-of-bias tool for randomized trials.

## Discussion

### Expanding the Role of Wearable Sensors in Health Monitoring

Wearable sensor technology, including IMU-based smartwatches, smart insoles, and multisensor systems, has significantly transformed health monitoring, rehabilitation tracking, and disease risk assessment [36]. These devices enable continuous, real-world tracking of mobility and functional health, addressing key limitations of traditional clinical assessments [37]. The reviewed studies highlight these devices’ diverse applications in neurological, musculoskeletal, cardiovascular, and metabolic conditions, supporting early disease detection, remote therapy adherence, and precision rehabilitation [38].

Observational studies accounted for 43% (13/30) of the reviewed studies, reflecting the feasibility of longitudinal monitoring, whereas experimental studies made up 27% (8/30), playing a crucial role in validating sensor-based assessments. However, the limited number of RCTs, representing only 10% (3/10) of the studies, underscores the need for rigorous intervention-based research to establish causal relationships between wearable sensor use and patient outcomes. The study populations were diverse, with healthy individuals comprising 33.3% of the participants, often included for sensor validation and reliability testing. Clinical populations, including individuals with PD, stroke, frailty, and cardiovascular conditions, were the primary focus of applied research, demonstrating the potential for wearable sensors to support patient management in real-world health care settings.

### Evolution of Wearable Sensor Applications

In our review, 30% (9/30) of the included studies conducted validation in healthy adults, indicating that early wearable-sensor research primarily focused on device feasibility and performance testing before expanding into clinical populations. These devices have revolutionized mobility monitoring, particularly in neurodegenerative conditions such as PD, MS, and stroke, where continuous tracking of gait parameters enables early detection of motor impairments and disease progression. They also play a significant role in frailty assessment (4/30, 13%) and fall risk prediction. Smart insoles demonstrate high predictive accuracy (AUROC=0.919; *P*<.05) as noninvasive, real-world mobility assessment tools.

### Technological Integration and Advances in Data Processing

The studies primarily used IMU-based systems (20/30, 67%) and smartwatches (8/30, 27%). Wrist-worn sensors were the most common, representing 43% (13/30) of the devices used, as they offer practicality, ease of wear, and convenience for everyday use. Ankle- and thigh-mounted sensors accounted for 23% (7/30) of applications and were primarily used for gait and posture assessments, whereas multisensor systems integrating electrocardiograms and pressure sensors provided additional biomechanical and cardiovascular insights, although they were less frequently studied.

Advances in ML have significantly enhanced data interpretation and predictive capabilities in wearable sensor applications [2]. Random forest models, applied in 20% (6/30) of the studies, were widely used for gait classification and activity recognition, whereas deep learning techniques were applied in 17% (5/30) of the studies and demonstrated high accuracy in long-term movement analysis. Elastic net regression and SVMs were used in 13% (4/30) of cases for classification tasks, whereas PCA was used in 7% (2/30) of the studies to reduce dimensionality and optimize data processing. However, variability in feature extraction methods remains a challenge. Standardized approaches are needed to improve reproducibility and clinical translation.

### Clinical Applications of Wearable Sensors

Wearable sensors demonstrated strong feasibility across multiple health care applications, including rehabilitation monitoring, disease risk assessment, activity tracking, and gait analysis. For rehabilitation assessment, wearable sensors improved postsurgical monitoring in patients who underwent knee arthroplasty, showing 52% better bone stimulus and 371% better impact load tracking. Wearable sensors for cardiac rehabilitation demonstrated reliable 6-minute walk distance prediction, with an error of 42.8 m and an *R*^2^ value of 0.661, facilitating remote patient monitoring. In stroke rehabilitation, IMU-based movement quality assessments achieved 92% accuracy (*F*_1_-score=0.95), supporting their use for personalized therapy and remote monitoring.

Recent studies have also extended the application of IMU-based wearable sensors to shoulder rehabilitation. Tranquilli et al [39] demonstrated that a single IMU could simultaneously capture joint mobility and muscle strength dynamics during postinjury recovery. Ajčević et al [40] applied IMU sensors to quantify shoulder kinematics and evaluate therapeutic response in adhesive capsulitis, whereas Parel et al [41] introduced a kinematic biofeedback program integrating inertial sensors for patients after rotator cuff repair. These studies highlight the versatility of IMU technology for upper-limb functional assessment and real-time feedback during rehabilitation.

Wearable sensors also played a key role in disease prediction and risk assessment. In PD monitoring, wearable technology achieved an accuracy of 83% in tremor detection and both a sensitivity and specificity of 86% in fall risk prediction, supporting the feasibility of early intervention strategies. Fall risk assessments using wearable sensors reached an AUROC value of 0.919, demonstrating their ability to provide noninvasive, real-world alternatives to clinical assessments such as the TUG and POMA tests.

Activity and behavior tracking applications showed high accuracy, particularly in arthritis-related pain and function monitoring, where significant correlations were observed between morning pain scores and 5-time sit-to-stand performance, with *P* values of less than .05. Consumer wearables such as Fitbit and iOS-integrated smartwatches achieved a strong agreement with research-grade sensors, with an AUROC of 0.93, demonstrating their feasibility for large-scale, real-world activity tracking. In medication adherence monitoring, smartwatch-based tracking achieved an accuracy of 93.6%, highlighting its potential for improving adherence in chronic disease management.

Gait and balance assessments using wearable sensors provided highly accurate insights into functional mobility. Head-worn IMU–based gait symmetry analysis reached an accuracy of 99.35%, indicating its effectiveness in neuromuscular rehabilitation and postural correction. Wearable sensor–based assessments of balance and mobility for patients with MS achieved an AUROC of 0.97, reinforcing their potential to support personalized rehabilitation planning and disease progression monitoring.

The included studies encompassed diverse populations, including healthy adults, neurological patients (eg, PD), individuals with musculoskeletal disorders, and pediatric or rehabilitation cohorts. This diversity introduces biomechanical and physiological variability in gait patterns, movement strategies, and sensor placement feasibility. Differences in muscle coordination, assistive device use, and experimental environments further contribute to heterogeneity. Given these variations, direct quantitative comparisons between studies were avoided. Instead, a narrative synthesis was used to identify overarching technological and methodological trends across populations. This approach emphasizes generalizable insights—such as the importance of standardized placement, calibration, and cross-population validation—while acknowledging disease-specific distinctions in biomechanics and sensor performance.

Many of the included studies (19/30, 63%) used ML algorithms such as random forest, deep learning, elastic net regression, and PCA for signal interpretation and disease classification. However, reporting transparency and methodological rigor varied substantially. Several studies (26/30, 87%) were limited by small sample sizes and internal validation only, increasing the risk of model overfitting. In addition, few studies (4/30, 13%) provided sufficient details regarding cross-validation protocols, feature selection strategies, or hyperparameter optimization.

Adherence to standardized ML reporting frameworks—such as the Transparent Reporting of a Multivariable Model for Individual Prognosis or Diagnosis–Artificial Intelligence and Prediction of Model Risk of Bias Assessment Tool–Artificial Intelligence—was rarely observed, which may affect reproducibility and generalizability.

Future research should emphasize external validation, open-source code sharing, and adherence to established ML reporting standards to ensure reliability and transparency in sensor-based clinical modeling.

### Challenges in Wearable Sensor Research

Despite the promising applications of wearable sensors, several challenges remain that must be addressed to ensure widespread clinical adoption and real-world impact.

First, small sample sizes (26/30, 87% of the studies) and limited real-world validation (12/30, 40% of the studies) reduced finding generalizability. Short study durations (8/30, 27%) also hindered long-term effectiveness assessment. Beyond these methodological limitations, the scope of this review was restricted to English-language, peer-reviewed publications, excluding gray literature such as conference abstracts and theses. This language restriction and publication bias may have favored studies reporting positive or statistically significant outcomes, potentially overestimating the clinical impact of wearable sensor technologies. Furthermore, although some studies (3/30, 10%) discussed the potential cost-effectiveness of sensor-based systems, no direct economic evaluations were identified, limiting the ability to substantiate financial feasibility claims.

Technical challenges also persist. Variability in signal-to-noise ratios, sensor displacement errors, and inconsistencies in data collection protocols underscore the need for improved hardware design and standardized preprocessing algorithms. Differences in feature extraction and model architectures limit cross-study comparisons and reproducibility.

Finally, the field urgently requires greater standardization. Variability in sensor placement, protocols, and data interpretation hinders reproducibility and large-scale comparison. Establishing consensus-driven guidelines for wearable sensor research—including standardized task protocols, data reporting frameworks, and model transparency criteria—will be essential to enable scalability, reproducibility, and eventual clinical translation.

### Future Directions

To fully realize the potential of wearable sensors in health care, future research should focus on several key areas. Expanding RCTs is essential to establish causal relationships between wearable sensor use and health outcomes beyond feasibility studies. Standardized data analysis frameworks will improve comparability and reproducibility, enabling integration into multicenter trials and large-scale studies. Long-term, multicenter studies will enhance real-world validation and assess sensor accuracy, usability, and adoption across health care settings.

Integration with cloud-based platforms and telemedicine will enhance scalability and enable real-time remote monitoring across diverse populations [42]. Cost-effectiveness analyses will determine financial feasibility and accessibility, supporting broader health care adoption and effective use in resource-limited settings [43].

### Conclusions

This systematic review highlights the growing clinical relevance of wearable sensors for rehabilitation monitoring, disease risk assessment, and personalized health care. IMU-based smartwatches, multisensor systems, and gait-monitoring devices demonstrate high accuracy in mobility assessment, fall risk prediction, and chronic disease management for digital health and precision medicine.

Despite their utility, the following challenges remain: small sample sizes, real-world validation gaps, and inconsistent ML methodologies. Future research should standardize protocols, expand clinical trials, and integrate sensors into telemedicine and cloud-based analytics platforms.

Overcoming these challenges will enable wearable sensors to revolutionize health care through real-time, noninvasive monitoring that bridges traditional clinical assessments and continuous real-world health tracking.

## Appendix

Multimedia Appendix 1 PRISMA 2020 checklist.

## Abbreviations

AUROC: area under the receiver operating characteristic curve
IMU: inertial measurement unit
JBI: Joanna Briggs Institute
ML: machine learning
MS: multiple sclerosis
NOS: Newcastle-Ottawa Scale
PCA: principal component analysis
PD: Parkinson disease
POMA: Performance-Oriented Mobility Assessment
PRISMA: Preferred Reporting Items for Systematic Reviews and Meta-Analyses
RCT: randomized controlled trial
RoB 2: version 2 of the Cochrane risk-of-bias tool for randomized trials
SVM: support vector machine
TUG: Timed Up and Go

## Data Availability

This systematic review paper does not include original data or materials as it primarily synthesizes and analyzes existing studies. Therefore, there are no specific datasets or materials available for sharing.
